# Editorial: Advancements in surgical strategies and technologies for cranial nerve disorders

**DOI:** 10.3389/fneur.2025.1661839

**Published:** 2025-08-07

**Authors:** Jun Zhong, Shiting Li

**Affiliations:** Department of Neurosurgery, Xinhua Hospital, Shanghai JiaoTong University School of Medicine, Shanghai, China

**Keywords:** trigeminal neuralgia, cranial nerve disorders, hyperexcitability, hemifacial spasm, microvascular decompression (MVD), percutaneous ballon compression, surgical strategy, minimal invasive operation

## Category

Cranial nerve disorders can generally be categorized into two principal classifications: hypofunctional states, which are defined by diminished or compromised neural activity, and hyperfunctional conditions, distinguished by exaggerated or inordinately intensified nerve reactions. The former group includes clinical presentations such as anosmia, visual impairment, external ophthalmoplegia, facial palsy, and facial anesthesia, *etc*. In contrast, the latter comprises well-known conditions such as hemifacial spasm, trigeminal neuralgia, glossopharyngeal neuralgia, and post-herpetic neuralgia following herpes zoster infection, *etc*. Hypofunctional states generally arise due to structural nerve damage, often induced by a variety of factors including schwannomas, traumatic injuries, or external compression from neighboring neoplasms, as well as inadvertent iatrogenic interventions.

## Mechanism

In hyperexcitability disorders of the cranial nerves, the key factor is that ectopic action potentials originate within the nerves themselves, rather than from the brainstem nuclei or ganglia (1). Under normal conditions, movements related to facial expressions are initiated by impulses generated in the cerebral cortex, whereas facial sensations are perceived through stimulation of sensory receptors located in the facial region. The most prevalent cause of hemifacial spasm (HFS) or trigeminal neuralgia (TN) is neurovascular conflict (2, 3). In patients with a constricted cerebellopontine angle (CPA), the proximity between cranial nerve roots and adjacent blood vessels gradually increases with age, ultimately exerting considerable pressure on the nerve (4). Due to the interfacial friction caused by the pulse, the nerve undergoes demyelination. When this pathological process advances to a certain stage, a cascade of signaling pathways mediated by inflammatory cytokines is triggered, ultimately leading to the expression of specific transmembrane proteins on the nerve surface (5, 6). These transmembrane proteins include mechanosensitive ion and voltage-gated channels. With pulsatile compressions, it drives the resting potential toward depolarization forming a state of subthreshold membrane potential oscillation. Under such precarious conditions, a precise mechanical stimulus may significantly lower the threshold required for membrane depolarization, thereby facilitating the activation of sodium channels and ultimately triggering propagatable action potentials. When these ectopic action potentials propagate to the nerve-muscle junctions, an attack of irregular muscle constriction occurs while to the central cortex, an illusion of sharp pain is perceived (1, 7–9, 21).

This etiological explanation clarifies why elderly individuals of Asian descent exhibit a heightened predisposition to conditions such as trigeminal neuralgia and hemifacial spasm. Due to distinct anatomical characteristics, the cerebellopontine angle in East Asians tends to be more compact compared to that of other racial groups. As aging progresses, the brain undergoes atrophy while blood vessels experience sclerosis, gradually drawing these structures into closer proximity within the confined CPA space (4).

This hypothesis effectively explains how emotional stimuli can trigger paroxysmal attacks. When individuals experience emotions such as nervousness or excitement, their heart rate and blood pressure undergo physiological changes. These changes generate a “precisely calibrated compression”—defined by specific frequency and amplitude that correspond to the individual's heartbeat and blood pressure—which acts upon the targeted nerve and triggers an impulse. In the absence of such pressure, the pulsative stimulus is effectively removed, thereby accounting for the prompt alleviation of symptoms frequently witnessed following a successful microvascular decompression (MVD) procedure (1). Postoperative recurrence may be attributed to improper placement of Teflon, which can induce granuloma formation, as the nerve root in TN patients remains highly vulnerable to neoplastic involvement as well as vascular compression (10). Besides, it may illustrate the clinical phenomenon that secondary TN or HFS cases are often caused by local epidermoid cysts or meningiomas rather than schwannomas perse (11, 12). In fact, due to their expansive growth pattern along the nerve sheath, schwannomas may act as an insulating barrier, protecting the nerve root from direct compression.

## Strategy

A clear and logically consistent understanding of the pathogenesis is essential for guiding effective treatment strategies. The principle of MVD is to carefully detach the offending vessel from the nerve root, rather than inserting Teflon material between them for insulation. A successful operation depends on adequate exposure of the affected cranial nerve root segment (3). In addition, the use of oversized or excessive Teflon felt should be avoided, as it may impair visualization and hinder accurate identification of the offending artery. Regardless of the vessel, neoplasm, or Teflon, any pulsatile contact may potentially trigger an ectopic impulse (10). A recommended approach involves initiating the dissection at the level of the caudal cranial nerves, followed by careful mobilization of the parent artery in a proximal and lateral direction, with small pieces of Geofoam strategically placed between the artery and the brainstem. By the time the facial nerve is reached, the offending vessel is often already displaced (13). Furthermore, safety considerations hold paramount importance in this functional neurosurgical procedure (14). The use of a sling technique within such a confined operative space is generally not suggested (1). Once an ideal leverage point for arterial displacement is identified, the decompression process becomes more efficient. It is essential to align the positioning of the artery with the natural direction of blood flow, ensuring long-term stability as the Geofoam is gradually resorbed, without causing recoil (15). In summary, the procedure should be performed using the most straightforward and time-efficient method available.

## Simplification

In addition to these classical techniques, simplified and minimally invasive approaches must not be overlooked. Our foremost priority lies in maximizing patient satisfaction rather than executing complex surgical interventions; thus, the procedure should be carried out expeditiously with minimal disruption to cerebral function. In recent years, percutaneous balloon compression (PBC) has gained increasing acceptance within the medical community (16). During inflation of the balloon with contrast agent in Meckel's cave, a pear-shaped opacity is typically observed on lateral fluoroscopy. This image consists of a rounded base oriented toward the semilunar ganglion, a central portion encompassing the rootlets, a head corresponding to the trigeminal root as it crosses the porus (neck), and a stalk directed toward the catheter tip. It is remarkable that the vast expanse of Meckel's cave is predominantly occupied by rootlets, with merely a small anterior portion housing the trigeminal ganglion (17). When a balloon is inflated within the cave, both the rootlets and the ganglion are subjected to compression, with the highest pressure concentrated at the narrowest zone through which the nerve fibers pass via the porus. A large-headed “pear” may indicate an increased risk of postoperative diplopia, facial hypoesthesia, and masticatory weakness due to possible damage to the abducent nerve or the trigeminal nerve fibers, including the motor branch. Considering that the rootlets consist of regenerative axons linked to peripheral nerves, while the ganglion houses neurons related to the non-renewable elements of the central nervous system, it becomes justifiable to apply targeted pressure on the ganglion as a preventive measure against recurrence (18, 19). Due to the structural distinctions between the slender, unmyelinated nociceptive neurons responsible for pain perception and the thicker, myelinated tactile neurons involved in touch sensation, precise control over the placement and timing of the PBC enables selective ablation of pain-transmitting nerves while largely sparing other sensory neural pathways (20).

## Summary

In conclusion, the future management of cranial nerve disorders should prioritize a deeper understanding of the underlying pathogenesis and embrace less invasive therapeutic strategies. Surgical techniques must be further refined to achieve greater simplicity and precision, with the ultimate vision being the successful treatment of these conditions through non-invasive or minimally invasive means.
